# Bias distribution and regulation in photoelectrochemical overall water-splitting cells

**DOI:** 10.1093/nsr/nwae053

**Published:** 2024-02-06

**Authors:** Kun Dang, Siqin Liu, Lei Wu, Daojian Tang, Jing Xue, Jiaming Wang, Hongwei Ji, Chuncheng Chen, Yuchao Zhang, Jincai Zhao

**Affiliations:** Key Laboratory of Photochemistry, CAS Research/Education Center for Excellence in Molecular Sciences, Institute of Chemistry, Chinese Academy of Sciences, Beijing 100190, China; University of Chinese Academy of Sciences, Beijing 100049, China; Key Laboratory of Photochemistry, CAS Research/Education Center for Excellence in Molecular Sciences, Institute of Chemistry, Chinese Academy of Sciences, Beijing 100190, China; University of Chinese Academy of Sciences, Beijing 100049, China; Key Laboratory of Photochemistry, CAS Research/Education Center for Excellence in Molecular Sciences, Institute of Chemistry, Chinese Academy of Sciences, Beijing 100190, China; University of Chinese Academy of Sciences, Beijing 100049, China; Key Laboratory of Photochemistry, CAS Research/Education Center for Excellence in Molecular Sciences, Institute of Chemistry, Chinese Academy of Sciences, Beijing 100190, China; University of Chinese Academy of Sciences, Beijing 100049, China; Key Laboratory of Photochemistry, CAS Research/Education Center for Excellence in Molecular Sciences, Institute of Chemistry, Chinese Academy of Sciences, Beijing 100190, China; University of Chinese Academy of Sciences, Beijing 100049, China; Key Laboratory of Photochemistry, CAS Research/Education Center for Excellence in Molecular Sciences, Institute of Chemistry, Chinese Academy of Sciences, Beijing 100190, China; University of Chinese Academy of Sciences, Beijing 100049, China; Key Laboratory of Photochemistry, CAS Research/Education Center for Excellence in Molecular Sciences, Institute of Chemistry, Chinese Academy of Sciences, Beijing 100190, China; University of Chinese Academy of Sciences, Beijing 100049, China; Key Laboratory of Photochemistry, CAS Research/Education Center for Excellence in Molecular Sciences, Institute of Chemistry, Chinese Academy of Sciences, Beijing 100190, China; University of Chinese Academy of Sciences, Beijing 100049, China; Key Laboratory of Photochemistry, CAS Research/Education Center for Excellence in Molecular Sciences, Institute of Chemistry, Chinese Academy of Sciences, Beijing 100190, China; University of Chinese Academy of Sciences, Beijing 100049, China; Key Laboratory of Photochemistry, CAS Research/Education Center for Excellence in Molecular Sciences, Institute of Chemistry, Chinese Academy of Sciences, Beijing 100190, China; University of Chinese Academy of Sciences, Beijing 100049, China

**Keywords:** photoelectrochemistry, overall reaction cell, bias distribution, photoanode, water splitting

## Abstract

The water oxidation half-reaction at anodes is always considered the rate-limiting step of overall water splitting (OWS), but the actual bias distribution between photoanodes and cathodes of photoelectrochemical (PEC) OWS cells has not been investigated systematically. In this work, we find that, for PEC cells consisting of photoanodes (nickel-modified *n*-Si [Ni/*n*-Si] and α-Fe_2_O_3_) with low photovoltage (*V*_ph_ < 1 V), a large portion of applied bias is exerted on the Pt cathode for satisfying the hydrogen evolution thermodynamics, showing a thermodynamics-controlled characteristic. In contrast, for photoanodes (TiO_2_ and BiVO_4_) with *V*_ph_ > 1 V, the bias required for cathode activation can be significantly reduced, exhibiting a kinetics-controlled characteristic. Further investigations show that the bias distribution can be regulated by tuning the electrolyte pH and using alternative half-reaction couplings. Accordingly, a volcano plot is presented for the rational design of the overall reactions and unbiased PEC cells. Motivated by this, an unbiased PEC cell consisting of a simple Ni/*n*-Si photoanode and Pt cathode is assembled, delivering a photocurrent density of 5.3 ± 0.2 mA cm^−2^.

## Teaser

This work reveals a general rule for measuring and describing the bias distribution in photoelectrochemical overall reaction cells, which inspires the rational design for high-performance unbiased photoelectrochemical cells in diversified practical scenarios.

## INTRODUCTION

The photoelectrochemical (PEC) overall water-splitting reaction (OWS) has been developed in recent decades; in particular, impressive achievements have been made on novel catalysts, characterization methods and reaction mechanisms [1–4]. Compared with the hydrogen evolution reaction (HER), the oxygen evolution reaction (OER) is deemed a bottleneck of OWS due to its sluggish kinetics [5,6]. Therefore, alternative oxidation half-reactions with better thermodynamics or kinetics have been explored, including alcohol [7,8], urea [6,9] and ammonia oxidation reactions [5,10], to lower the applied bias on the anode. With alternative half-reactions, the ultimate goal is to fabricate high-performance unbiased two-electrode cells [11]. However, researchers mostly focus on the performance of the working electrode of a three-electrode cell, largely ignoring the polarization process of the counter electrode [12]. The synergistic mechanism between the oxidation half-reaction at the anode and the reduction half-reaction at the cathode remains elusive.

Recently, PEC overall reactions with various half-reaction couplings have been gaining attention [13–20]. For example, glucose oxidation or C–H halogenation coupling with the HER [16,20] and biomass or glycerol oxidation coupling with the CO_2_ reduction reaction (CO_2_RR) [13,17] have been investigated to reduce the overall bias consumption. In the design of a PEC overall reaction cell with matched half-reaction thermodynamics and kinetics, the bias consumption of the anode and the cathode under operating conditions should be discerned [21]. Thermodynamic analysis shows theoretically that 90% of the electricity input is consumed via the OER for the coupling of the OER and CO_2_RR [22]. However, to the best of our knowledge, the actual bias distribution between photoanodes and cathodes in PEC overall reaction cells has rarely been experimentally explored.

**Figure 1.**
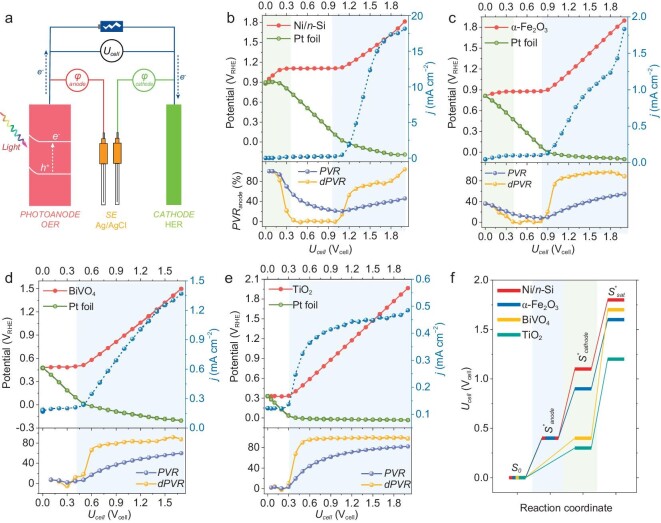
Measured bias distribution of PEC OWS cells with various photoanodes. (a) Schematic of bias distribution measurements. The cell voltage (*U*_cell_) was controlled and the photoanode or cathode potentials were recorded versus the sensing electrode (SE, Ag/AgCl). Steady current density, bias distribution and corresponding partial voltage ratio (*PVR*) of photoanodes (*PVR*_anode_) as a function of cell voltage with different photoanodes: (b) Ni/*n*-Si, (c) α-Fe_2_O_3_, (d) BiVO_4_ and (e) TiO_2_. Most measurements were performed in 1 M NaOH under AM 1.5 G illumination, except for the BiVO_4_ (0.2 M potassium borate buffer, pH = 9). (f) Reaction coordinates of OWS in PEC cells consisting of various photoanodes and Pt cathode. *S*_0_, S*_anode_, S*_cathode_ and S*_sat_ correspond to the initial state, OER onset state, HER onset state and the state reaching a saturation photocurrent of the PEC OWS cell, respectively. The data acquisition process in this figure was conducted using a two-electrode single-chamber PEC cell.

Here, we develop an experimental method for measuring the bias distribution in a two-electrode PEC cell (Fig. 1a). A systematic investigation of the bias distribution between various representative photoanodes and a Pt cathode reveals that the bias consumption of the electrodes depends on the photovoltage (*V*_ph_) of the photoanode and the Fermi level (*E*_f_) of the cathode. Taking the Ni/*n*-Si photoanode as the model, the bias distribution is regulated by tuning the electrolyte pH and using alternative half-reaction couplings. A volcano plot is depicted to propose a descriptor for evaluating compatibility between various half-reactions, pointing towards a general method of designing high-performance PEC overall reaction cells. Then an unbiased PEC cell consisting of a simple Ni/*n*-Si photoanode and a Pt cathode is achieved, delivering a photocurrent of 5.3 ± 0.2 mA cm^−2^.

## RESULTS AND DISCUSSION

### Bias distribution of representative photoanodes

The bias distribution measurements were conducted in a two-electrode PEC cell with Pt metal as the cathode, coupled with various photoanodes (Fig. 1a). For comparison, the linear sweep voltammetry curves of these photoanodes in a three-electrode cell are exhibited in Fig. S1. The bias distribution profiles of the PEC OWS cells are presented in Fig. 1b–e. The bias applied to the two-electrode cell is denoted as *U*_cell_ in units of V_cell_, while the unit of the electrode potential is represented as V_RHE_. For the initial position (*U*_cell_ = 0 V_cell_) of the bias distribution profiles, the photoanode potential was equal to that of the cathode, corresponding to the short-circuit potential (*φ*_sc_) of the PEC cell. The ‘short circuit’ means no external bias is applied to a PEC device and the two electrodes are connected by a wire in ohmic contact, differing from the condition for open-circuit potential (OCP) measurements in three-electrode cells. The *φ*_sc_ reflects an initial equilibrium state in Fermi levels of the whole PEC cell. Its position is related to the conduction bands of *n*-type photoanodes but is affected by the quasi-Fermi-level pinning of photoanodes with abundant surface states. Therefore, a good linear relationship between *φ*_sc_ and *V*_ph_ was observed, as the measured *V*_ph_ was also influenced by the Fermi level pinning effect (additional details in Fig. S2). The bias distribution between various electrodes was quantificationally described using the partial voltage ratio (*PVR*) and the differential *PVR* (d*PVR*) of the photoanode (or cathode), as follows:


(1) \begin{eqnarray*}
PV{{R}_{{\mathrm{anode}}}}\!\left( \% \right){\mathrm{\ }} = {\mathrm{\ }}\frac{{{{\varphi }_{{\mathrm{anode}}\ }} - \ {{\varphi }_{{\mathrm{sc}}}}\ }}{{{{\varphi }_{{\mathrm{anode}}\ }} - \ {{\varphi }_{{\mathrm{cathode}}}}}}{\mathrm{\ }} \times {\mathrm{\ }}100{\mathrm{\% }},
\end{eqnarray*}

(2) \begin{eqnarray*}
{\mathrm{d}}PV{{R}_{{\mathrm{anode}}}}\!\left( \% \right){\mathrm{\ }} = {\mathrm{\ }}\frac{{\partial {{\varphi }_{{\mathrm{anode}}}}}}{{\partial {{U}_{{\mathrm{cell}}}}}}{\mathrm{\ }} \times {\mathrm{\ }}100{\mathrm{\% }}.
\end{eqnarray*}

where *PVR*_anode_ reflects the proportion of the overall bias consumed by the photoanode to shift its potential relative to the initial potential (*φ*_sc_) and d*PVR*_anode_ is the change in the bias distribution of the photoanode in each voltage-sweeping step. The value of *U*_cell_ equals the difference between *φ*_anode_ and *φ*_cathode_, as the ohmic drop can be ignored in the PEC tests on the studied single-chamber cell (Fig. S3).

Taking Ni/*n*-Si photoanode as an example, the bias distribution profiles displayed three regimes, as shown in Fig. 1b. Below 0.4 V_cell_ (Regime I), most of the applied bias (>50%) was used to charge the photoanode (shifting the photoanode potential from 0.89 to 1.11 V_RHE_). In this case, the OWS reaction was not triggered despite the Ni/*n*-Si photoanode having reached the OER onset potential (Fig. S1a), as the HER thermodynamics (<0 V_RHE_) was not satisfied by the Pt cathode. This resulted in the subsequent plateau regime of the photoanode potential. From 0.4 to 1.0 V_cell_ (Regime II), almost 100% of the increased bias was exerted on the cathode according to the d*PVR*_anode_–*U*_cell_ curve (bottom of Fig. 1b), while the photoanode potential remained at ∼1.11 V_RHE_ until the cathode potential enabled the HER thermodynamics. As the bias exceeded 1.0 V_cell_ (Regime III), the OWS reaction was triggered, accompanied by simultaneous shifts in the photoanode and cathode potentials. Finally, ∼40% of the overall bias was consumed (denoted as *PVR*_sat_, meaning the photoanode *PVR* achieving the saturation photocurrent) by the Ni/*n*-Si photoanode when the current density reached saturation at ∼1.8 V_cell_. Thus, a large portion of the applied bias was consumed by the Pt cathode in the PEC cell.

The bias distribution profiles of the α-Fe_2_O_3_ photoanode also displayed three distinct regimes (Fig. 1c). At 0.4 V_cell_, ∼16% of the bias was used to charge the surface-active species of the photoanode, as α-Fe_2_O_3_ undergoes a surface hole-trapping process before driving water oxidation [23–25]. From 0.4 to 0.8 V_cell_, almost 100% of the increased bias was used to charge the cathode (d*PVR*_anode_ in Fig. 1c). It is the insufficient HER thermodynamics of the cathode that accounted for the plateau photoanode potential in this regime. Finally, ∼44% of the overall bias was consumed by the α-Fe_2_O_3_ photoanode when it achieved the saturation photocurrent at 1.6 V_cell_. Another reported photoanode undergoing the surface-trapped hole mechanism is the plasmonic Au/TiO_2_ photoanode [26,27], whose bias distribution properties are shown in Fig. S4a. In that case, the charging of the anode consumed most of the applied bias, which was consistent with its sluggish OER kinetics (Fig. S4b).

For the Ni/*n*-Si, α-Fe_2_O_3_ and plasmonic Au/TiO_2_ photoanodes, the applied bias was initially used to charge the surface-active species. As for the photoanodes undergoing direct hole transfer from the valence bands, namely BiVO_4_ [28] (Fig. 1d) and TiO_2_ [29] (Fig. 1e), the photoanode charging regime was skipped; one regime was retained for cathode charging (0–0.4 V_cell_ for BiVO_4_ and 0–0.3 V_cell_ for TiO_2_) and another was retained for OWS (beyond 0.4 and 0.3 V_cell_ for BiVO_4_ and TiO_2_, respectively). For BiVO_4_ at a bias of 0–0.4 V_cell_, nearly 100% of the bias was consumed by the cathode, as the OWS reaction was hindered by the HER thermodynamics. As for TiO_2_, almost all the bias below 0.3 V_cell_ was used to enable the HER. Due to the larger *V*_ph_ and more negative *φ*_sc_ of BiVO_4_ and TiO_2_, their bias consumption for the HER thermodynamics was greatly reduced compared with those of the PEC cells with Ni/*n*-Si and α-Fe_2_O_3_ photoanodes. Nevertheless, compared with the Pt cathode, BiVO_4_ and TiO_2_ consumed more bias at their saturation photocurrents, displaying ∼60% and ∼71% *PVR*_sat_, respectively, which implied that the OER was a rate-limiting step for these PEC cells. Considering the continuous oxygen evolution at the photoanodes, the oxygen in the electrolyte was not purposely removed in the above measurements to obtain more practical results. As a special case, control experiments (with oxygen removal) were conducted, as shown in Figs S5 and S6, with the Ni/*n*-Si and TiO_2_ photoanodes as examples.

Based on the abovementioned bias distribution measurements, two types of activation pathways of the PEC OWS cells are summarized in Fig. 1f. The key difference between them was the activation step for the photoanodes, which was closely related to the hole-transfer mechanism for the OER. For the photoanodes with *V*_ph_ of <1 V, namely α-Fe_2_O_3_ and Ni/*n*-Si, the HER was the thermodynamical limiting step, as S*_cathode_ consumed considerable bias. However, for the photoanodes with *V*_ph_ of >1 V, namely TiO_2_ and BiVO_4_, the process from S*_cathode_ to S*_sat_ consumed the most bias, which was ascribed to their inferior OER kinetics. Previous studies in three-electrode cells consider the OER as a bottleneck in OWS, whereas our results showed that the HER could also be a rate-limiting factor of OWS in practical two-electrode PEC cells. The mismatch between the OER and HER motivated us to regulate the bias distribution further.

**Figure 2.**
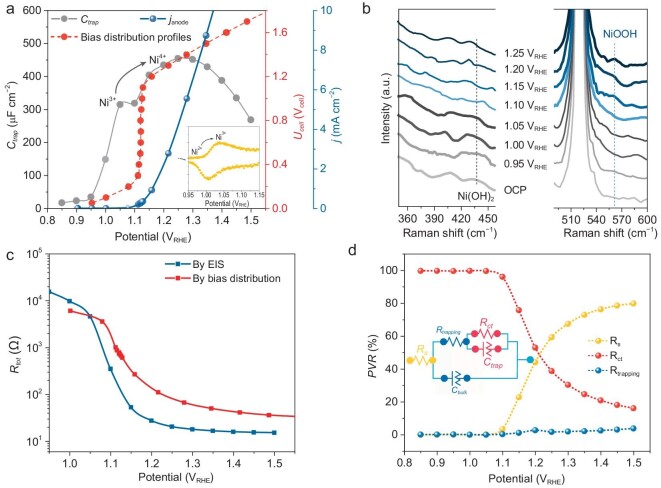
Characterizations of surface states. (a) Trap-state capacitance (*C*_trap_) extracted from electrochemical impedance spectroscopy (EIS), steady current density (*j*_anode_), bias distribution profiles of Ni/*n*-Si photoanode and cyclic voltammetry curves (inset) of the Ni/*n*-Si photoanode. Apart from the bias distribution measurements, all the data in this figure were obtained using a three-electrode cell. (b) *In situ* PEC Raman spectra of Ni/*n*-Si during the OER in 1 M NaOH in a three-electrode cell. (c) Comparison of *R*_tot_ obtained from EIS and bias distribution measurement. (d) Bias distribution on the Ni/*n*-Si photoanode at the level of circuit elements (inset: equivalent circuit model).

The following part focuses on the Ni/*n*-Si photoanode for further investigation. The surface-active species of the Ni/*n*-Si photoanode before and during the OER were investigated through potential-dependent electrochemical impedance spectroscopy (EIS) to understand the three regimes of the bias distribution profiles of this photoanode (Fig. 1b). Figure 2a shows two trap-state capacitance (*C*_trap_) peaks (1.05 and 1.25 V_RHE_, as shown by the gray dotted line), implying the involvement of two types of surface species in the OER process of the Ni/*n*-Si photoanode. The first peak (at 1.05 V_RHE_) was attributed to the accumulation of Ni^3+^ (NiOOH), as its potential regime overlapped with the redox peak of Ni^2+^/Ni^3+^ (inset of Fig. 2a) [30,31], which was contained in Regime I (Fig. 1b). The second peak (at 1.25 V_RHE_) corresponded to the transformation of Ni^3+^ to Ni^4+^, which served as a vital intermediate for the OER [32,33].

*In situ* PEC Raman spectra further confirmed the surface-active species (Fig. 2b and Fig. S7). Although Ni-based electrocatalysts have been widely studied [34–36], the circumstances of such catalysts when loaded on Si photoanodes remain to be explored. In Fig. 2b, the highest peak (at ∼520 cm^−1^) originated from the Si substrate, which overlapped with the signal of Ni^2+^–O [37]. The signal peak at 437 cm^−1^ was ascribed to the Ni(OH)_2_ [33,38], which appeared at the OCP; this suggested that most of the metal Ni was transformed into Ni(OH)_2_ or NiO during the pre-activation process (Figs S8 and S9). The intensity of this peak (at 437 cm^−1^) started to decay when the applied potential exceeded 1.0 V_RHE_, indicating the transformation of Ni^2+^/Ni^3+^, as revealed via EIS. Accordingly, the NiOOH peak observed at 560 cm^−1^ from ∼1.15 V_RHE_ acted as the real active species for OER in Regimes II and III (Fig. 1b).

The total resistance of the photoanode (*R*_tot_) was obtained by summarizing series resistance (*R*_s_), trapping resistance (*R*_trapping_) and charge transfer resistance (*R*_ct_) (*R*_tot_ = *R*_s_ + *R*_trapping_ + *R*_ct_) [23], as shown in Fig. 2c. Through Ohm's law, *R*_tot_ can also be calculated as follows:


(3) \begin{eqnarray*}{{R}_{{\mathrm{tot}}}}\! \left( {\mathrm{\Omega }} \right) = \frac{{{{\varphi }_{{\mathrm{anode}}}}\! \left( {{{{\mathrm{V}}}_{{\mathrm{RHE}}}}} \right){\mathrm{\ }} - {\mathrm{\ }}{{\varphi }_{{\mathrm{sc}}}}\!\left( {{{{\mathrm{V}}}_{{\mathrm{RHE}}}}} \right)}}{{I{\mathrm{ }}\!\left( {\mathrm{A}} \right)}}.
\end{eqnarray*}

The *R*_tot_ obtained from the EIS and bias distribution measurements showed a decreasing tendency from 1.05 V_RHE_, where the surface-active species (NiOOH) started to accumulate, as depicted in Fig. 2a and b. The shape similarity of the two potential-dependent *R*_tot_ curves enabled us to focus more details on the bias distribution at the electrolyte/photoanode interface based on the proportions of different resistance values.

At <1.05 V_RHE_, *R*_ct_ consumed almost 100% of the partial voltage on the photoanode (Fig. 2d), as the Ni(OH)_2_ on the surface of the Si photoanode is highly resistive [30,39], indicating that the applied bias mainly dropped at the photoanode/electrolyte interface. Under this condition, the high *R*_ct_ could be simplified as an open circuit; that is, the photoanode/electrolyte interface played the part of capacitance. The potential drop exerted on the *C*_trap_ promoted NiOOH accumulation, which corresponded to the activation process of the photoanode in Regime I (Fig. 1b). Furthermore, the high resistance of Ni(OH)_2_ led to a more distinct Regime I for photoanode charging compared with α-Fe_2_O_3_; ∼53% of the bias was consumed by Ni/*n*-Si in Regime I whereas only ∼16% of the bias was consumed in the case of α-Fe_2_O_3_ (Fig. 1b and c). When the potential of the photoanode shifted more positively than 1.10 V_RHE_, the *PVR* for *R*_trapping_ and *R*_s_ increased gradually, corresponding to the reduction in *R*_ct_ that was related to the transformation of Ni(OH)_2_ into NiOOH. This stage elucidates the bias distribution characteristics of the Ni/*n*-Si photoanode of the two-electrode PEC OWS cell. The following sections discuss the regulation of the bias distribution.

### Bias regulation through electrolyte pH

**Figure 3.**
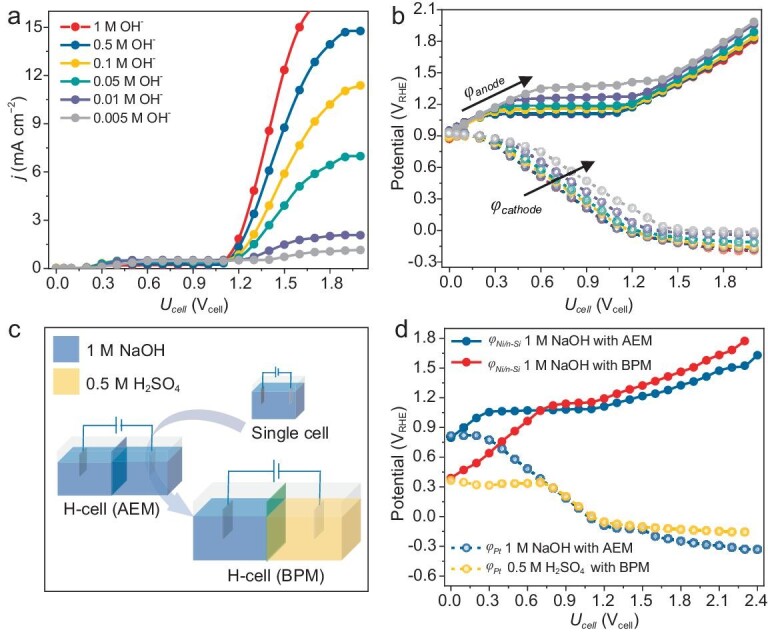
Bias regulation through pH of electrolyte in two-electrode PEC cell. (a) pH-dependent steady-state *J*–*V* curves and (b) corresponding bias distribution profiles of Ni/*n*-Si photoanode and Pt cathode in two-electrode single-chamber cell. (c) Schematic of pH regulation of electrolyte. (d) Bias distribution profiles of Ni/*n*-Si photoanode and Pt cathode in two-electrode H-cell with different catholyte pH values. Hereinafter, unless otherwise specified, a bipolar membrane (BPM) was used in the H-cell at a reverse bias to maintain the pH difference between the two chambers. For comparison, an H-cell separated by an anion-exchange membrane (AEM) was also evaluated.

As OWS is pH-sensitive, we attempted to regulate the bias distribution by tuning the electrolyte pH of the single-chamber PEC cell. Figure 3a presents the *J*–*V* curves of the Ni/*n*-Si photoanode for OWS at different concentrations of OH^−^ ([OH^−^]). With varying [OH^−^] from 1 to 0.005 M, the photocurrent decreased by over one order of magnitude. This decrease was related to the shallow valence band of the Ni/*n*-Si photoanode (Fig. S10), which made it more conducive thermodynamically to the oxidation of OH^−^ than H_2_O molecules. The influence of surface protonation was not a main factor for the pH-dependent performance in this work (additional details in the supplemental discussion of Fig. S10). Meanwhile, the bias distribution profiles of both the photoanode and cathode shifted positively with a decrease in the [OH^−^] (Fig. 3b), as the formation of NiOOH in Regime I and the OER kinetics were restrained at lower pH values. Although the *PVR*_sat_ of the Ni/*n*-Si photoanode increased slightly (by ∼5%) (Fig. S11a and b), enhancing the *PVR*_sat_ at the expense of the OER activity was pointless.

Measuring the bias distribution in two-electrode PEC cells enables the quantitative determination of the bias-determining step, thus providing valuable insights for optimizing the overall reaction. As the Pt cathode consumed more bias, we facilitated the HER thermodynamics by situating the photoanode and cathode under different pH conditions (0 and 13.6). The reaction was conducted in an H-cell separated by a bipolar membrane (BPM) (Fig. 3c), where 0.5 M H_2_SO_4_ and 1 M NaOH served as the catholyte and anolyte, respectively. For comparison, we measured the bias distribution using the same H-cell but separated by an anion-exchange membrane (AEM); 1 M NaOH was the electrolyte in both anode and cathode chambers (Fig. 3c). As shown in Fig. 3d, for the BPM cell, *φ*_sc_ presented a significantly negative shift from 0.81 to 0.39 V_RHE_, thus enhancing bias consumption of the photoanode to satisfy the OER thermodynamics. Accordingly, *PVR*_sat_ increased significantly to 71% (Fig. S11d) due to the good HER thermodynamics and kinetics in the catholyte with the optimal pH, which helped increase the photocurrent relative to that of the AEM cell (Fig. S11c).

### Bias regulation via alternative oxidation reaction

**Figure 4.**
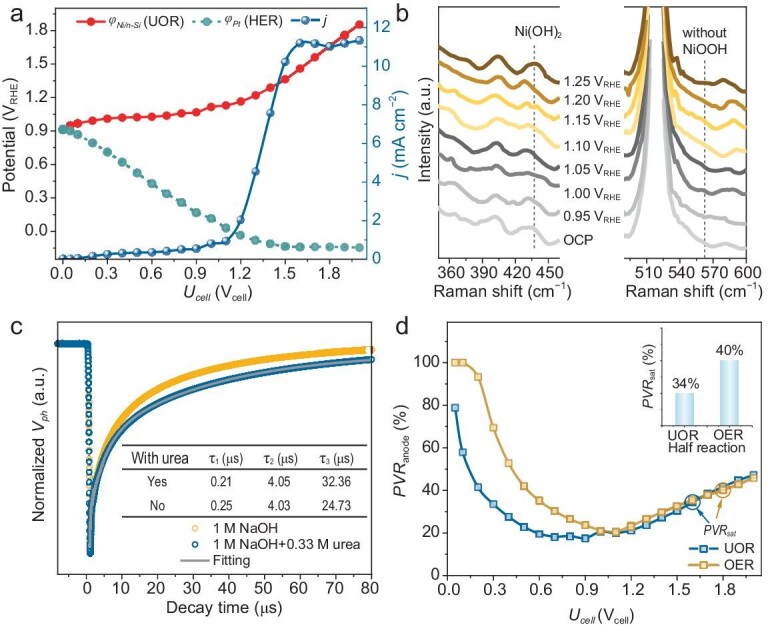
Bias regulation through urea oxidation reaction (UOR). (a) Bias distribution profiles and steady-state *J*–*V* curves of PEC cell with UOR as oxidation half-reaction in single-chamber two-electrode cell. (b) Potential-dependent *in situ* PEC Raman spectra of Ni/*n*-Si during UOR process in three-electrode Raman cell. (c) Transient photovoltage (TPV) spectra of Ni/*n*-Si photoanode in 1 M NaOH with or without 0.33 M urea at OCP. (d) Comparison of *PVR*_anode_ and *PVR*_sat_ (inset) during the OER and UOR at different biases.

We regulated the bias distribution using an alternative half-reaction. The urea oxidation reaction (UOR) has been widely studied due to its abundant applications in fuel cells, urea-rich wastewater treatment and other fields [6,9,40]. As the UOR has a lower thermodynamic redox potential than the OER, we attempted to regulate the bias distribution by coupling the UOR with the HER. As shown in Fig. 4a, the UOR||HER cell presented remarkably enhanced PEC activity, shifting the onset bias (*j*_onset_ = 0.5 mA cm^−2^) from 1.1 V_cell_ for OWS to 0.9 V_cell_, as the Ni^3+^ formed at 0.9 V_cell_ (1.05 V_RHE_ for the photoanode) enabled the UOR [41]. This was further confirmed via *in situ* PEC Raman measurements (Fig. 4b and Fig. S12). Compared with that under the OER process, the Ni^2+^ signal under the UOR remained at higher potentials (>1.05 V_RHE_), implying the rapid depletion of the formed Ni^3+^. Thus, the Ni^3+^ signals were not observed during the UOR process. According to the EIS results, the UOR began at 0.90 V_RHE_, as the high-frequency peak rose under such conditions (Fig. S13a), whereas the OER was not triggered until 1.10 V_RHE_ (Fig. S13b).

In addition, as revealed by the transient photovoltage (TPV) spectra (Fig. 4c), the decay half-time of photogenerated holes was longer (32.36 μs) than that for the urea-free cell (24.73 μs), which promoted the reaction kinetics at the photoanode. As a result, a remarkable decrease in the *PVR*_anode_ was observed below the bias of 1.0 V_cell_ (Fig. 4d). Particularly, compared with that under the OER, *PVR*_sat_ declined from 40% to 34% (inset of Fig. 4d), showing a larger bias consumption for the HER. Meanwhile, the bias needed to achieve the saturation photocurrent of the Ni/*n*-Si photoanode for the UOR reduced from 1.8 to 1.6 V_cell_. Therefore, regulating the bias distribution in PEC overall cells using alternative half-reactions is feasible.

### Summary of bias distributions

Based on the obtained bias distribution characteristics, we examined the feasibility of regulating the bias distribution in a PEC OWS cell by screening photoanode materials, tuning the electrolyte pH and using the alternative half-reaction couplings. To describe the compatibility between the half-reactions in a PEC overall reaction cell, we define a descriptor called the deviation degree (*η*), namely the degree to which *PVR*_sat_ deviates from a balanced bias distribution (50%), as follows:


(4) \begin{eqnarray*}
\eta {\mathrm{\ }} = \ \frac{{PV{{R}_{{\mathrm{sat}}\ }}\ }}{{100{\mathrm{\% }}}}{\mathrm{\ }} - {\mathrm{\ }}0.5.
\end{eqnarray*}

**Figure 5.**
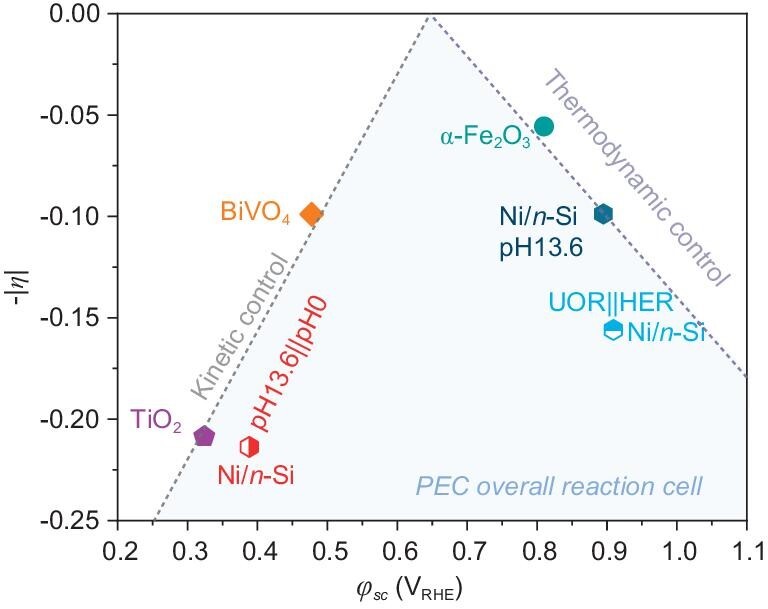
Summary of bias distributions. Volcano plot of deviation degree (*η*) with *φ*_sc_ of PEC overall reaction cells. The hexagonal points represent the Ni/*n*-Si photoanode under various conditions.

The relationship between *η* and the *φ*_sc_ of the PEC overall reaction cells is depicted in Fig. 5. For the points on the left side (TiO_2_ and BiVO_4_), the large *V*_ph_ (>1 V) shifted the *φ*_sc_ closer to the redox potential of H^+^/H_2_, greatly reducing the bias required for cathode charging, but the inferior OER kinetics of TiO_2_ and BiVO_4_ was a rate-limiting factor. These photoanodes exhibited kinetics-control characteristics. By contrast, for the points on the right side (Ni/*n*-Si and α-Fe_2_O_3_), the low *V*_ph_ (<1 V) resulted in a more positive *φ*_sc_. Thus, thermodynamic-controlled characteristics were observed; the cathode consumed a large amount of the applied bias to satisfy the HER thermodynamics, thus compensating for the sluggish OER kinetics. When both half-reactions were in their optimal pH environments (1 M NaOH||0.5 M H_2_SO_4_), the high value of *η* (∼0.21) agreed with the kinetics mismatch between the HER and OER half-reactions. Although *η* could be reduced to ∼0.10 via pH adjustment (both the photoanode and the cathode were under alkaline conditions), doing so was meaningless because it would have compromised the HER activity.

The volcano plot in Fig. 5 provides guidance for the rational design of PEC overall reaction cells. For example, in the assembly of a PEC cell for hydrogen production, semiconductors with a large *V*_ph_ (such as TiO_2_ and BiVO_4_) should be utilized as the photoanode to reduce the onset voltage of the overall reaction. Meanwhile, in addition to the thermodynamics of the alternative oxidation reaction, the kinetics should be considered to help reduce the overall bias needed to reach the saturation photocurrent. For assembling PEC cells for high-value oxidation reactions, photoanodes such as α-Fe_2_O_3_ and Ni/*n*-Si should be used, coupled with an alternative reduction reaction with more positive redox potentials. Although the HER is important for green energy production, coupling suitable reduction reactions with valuable oxidation reactions may be more economical. Particularly, if the onset potential of the selected reduction reaction is more positive than the *φ*_sc_ of a PEC OWS cell, then an unbiased PEC cell can be achieved without using photocathodes, thereby significantly reducing the cost of electric power. Under such conditions, the photogenerated electrons from the conduction bands of the photoanode can be injected spontaneously into the oxidizing agents on the cathode. The consumption of photogenerated electrons inhibits their recombination with holes, thereby simultaneously promoting the oxidation reaction at the photoanode, and producing a continuous current flow throughout the PEC cell. Furthermore, as seen in Fig. 5, *φ*_sc_ can be regulated by the *V*_ph_ of photoanodes, the electrolyte pH and alternative half-reactions, hence improving the flexibility in the fabrication of unbiased PEC cells.

### Unbiased PEC cell fabrication scheme inspired by the bias distribution investigation

**Figure 6.**
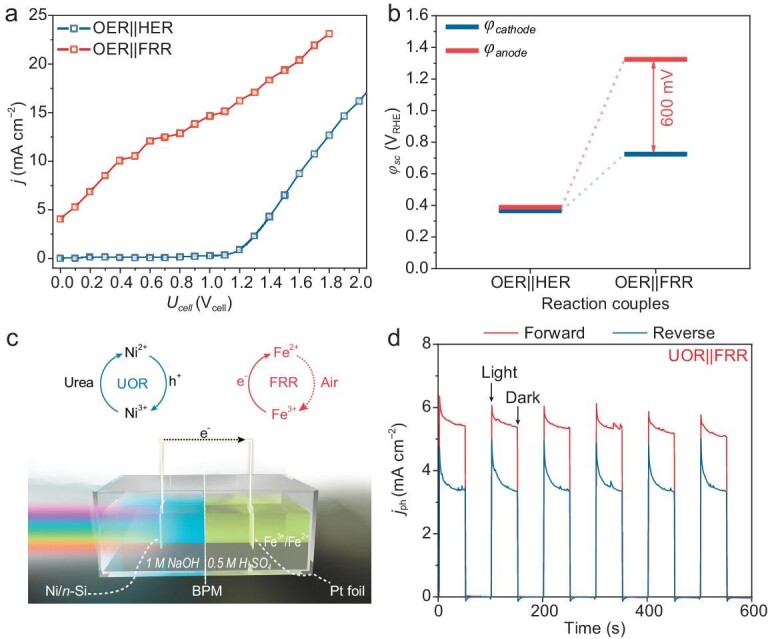
Unbiased PEC cells designed according to bias distribution measurements. (a) Steady-state *J*–*V* curves and (b) *φ*_sc_ of BPM-separated two-electrode PEC cell consisting of Ni/*n*-Si photoanode and Pt cathode for different half-reaction couplings (OER||HER and OER||FRR). (c) Schematic of unbiased PEC cell for urea removal and (d) unbiased *I*–*t* profiles of UOR||FRR couplings. The words ‘reverse’ and ‘forward’ refer to the BPM used at reverse and forward biases, respectively (Fig. S15). Here, 0.5 M Fe(NO_3_)_3_ was dissolved in a 0.5 M H_2_SO_4_ solution in the cathode chamber of a BPM-separated H-cell, and the anolyte was 1 M NaOH.

Given the above volcano plot analysis, with the Ni/*n*-Si photoanode and the Pt cathode, we assembled an unbiased PEC cell by replacing the HER with the Fe^3+^ reduction reaction (FRR). We selected the Fe^3+^/Fe^2+^ redox couple due to its more positive redox potential (0.77 V_NHE_) compared with HER and the *φ*_sc_ of the Ni/*n*-Si(pH = 13.6)||Pt(pH = 0) PEC OWS cell. Besides, the Fe^3+^/Fe^2+^ redox couple could feasibly decouple the OER and HER, thus greatly reducing the overall bias needed for electrocatalytic HER [42–44]. Moreover, the FRR product (Fe^2+^) is an essential reagent in the Fenton reaction, which is widely used for wastewater treatment [45,46]. For the PEC OWS cell, a bias of ∼1.2 V_cell_ was needed to trigger the overall reaction (Fig. 6a, Figs S14 and S15). As for the coupling of OER and FRR, an unbiased PEC cell was achieved; it exhibited a high photocurrent density of 3.2 ± 0.1 mA cm^−2^ without any tandem photocathode or photovoltaics (Fig. 6a). The Fe^3+^ ions served as electron acceptors, which consumed the photogenerated electrons from the conduction band of the photoanode, reducing the carrier recombination. Thus, the generation of surface-active species of the Ni/*n*-Si photoanode was promoted simultaneously, resulting in the split of *φ*_sc_ with a potential difference of ∼600 mV (Fig. 6b). This splitting provides the driving force needed to generate photocurrents under short-circuit conditions without an external bias.

In addition, an unbiased PEC cell for urea oxidation was assembled through the coupling of UOR||FRR (Fig. 6c); it exhibited a photocurrent density of 3.4 ± 0.1 mA cm^−2^ with a reverse BPM and 5.3 ± 0.2 mA cm^−2^ with a forward BPM (Fig. 6d). This further demonstrated the effectiveness of regulating the bias distribution via alternative half-reactions. The forward BPM (Fig. S14) adopts electrochemical neutralization energy [47,48] and therefore shows a higher photocurrent density. Notably, the photoanode used here, Ni/*n*-Si, is not optimal for the UOR compared with previously reported photoanodes [40,49]. However, it was chosen as the model photoanode due to its simple, well-defined nature. In other words, the performance of such a PEC cell could be further enhanced using more advanced catalysts, thereby leading to greater economic benefits for the practical applications of such cells. The UOR can also be replaced by olefin epoxidation, chlorine alkali industry and other useful oxidation reactions, covering more unbiased PEC cells in various scenarios and options in the future.

## CONCLUSION

In summary, we propose a bias distribution measurement method for estimating the bias consumption of various half-reactions in two-electrode PEC overall reaction cells under operating conditions. Through a systematic bias distribution investigation, we show that the HER can also be a rate-limiting factor in practical PEC OWS cells, which underscores the need to decouple the HER and OER, and increase focus on optimizing reductive half-reactions. We also present three general ways to regulate the bias distribution: using photoanodes with appropriate *V*_ph_, tuning the pH of the electrolyte and using alternative half-reaction couplings. Accordingly, a volcano plot is depicted to provide guidance for the rational design of overall reactions and unbiased PEC cells in diverse practical scenarios. Driven by these findings, we assemble a high-performance unbiased PEC cell consisting of a simple Ni/*n*-Si photoanode and a Pt cathode, which can be valuable for wastewater treatment and artificial photosynthesis.

## METHODS

### PEC measurements

Most PEC measures were performed on the PGSTAT302N (Autolab, Metrohm) electrochemical workstation in a 50-mL single-chamber cell with a circulator bath of 25°C, under the illumination of a Xenon lamp (100 mW cm^−2^) equipped with an AM 1.5 G filter. For the two-electrode bias distribution measurements, the Ag/AgCl electrode (filled by saturated KCl solution) was connected to the PX1000 model of the Autolab workstation to monitor the potentials of the photoanode or cathode. The interval of the applied overall bias was set at 0.1 V beginning with 0 V_cell_, and the steady *I*–*t* measurements were carried out with 100–300 s of duration time for each bias. The measured short-circuit potential at 0 V_cell_ usually displayed a bit of fluctuation for various photoanodes, so the steady potential of photoanodes and cathode at 0 V_cell_ was recorded more than three times. Meanwhile, according to the data fluctuation degree under 0 V_cell_ for different photoanodes, a point between 0 and 0.1 V_cell_ was inserted into the bias distribution profiles. For most cases, the inserted data point was measured under 0.05 V_cell_, while it was 0.001 V_cell_ for α-Fe_2_O_3_. For the pH effect measurements, the ionic strength of all the solutions with different OH^−^ concentrations was maintained at 1 M by adding NaClO_4_ electrolyte. Potentials were converted into the reversible hydrogen electrode (RHE) scale according to the Nernst equation: *E*_RHE_ = *E*_Ag/AgCl_ + 0.059 × pH + 0.197. For the PEC measurements in an H-cell, the potential of the cathode in acid catholyte (pH = 0) was calibrated to the Hg/Hg_2_SO_4_ (filled by saturated K_2_SO_4_ solution) electrode (CHI 151, CH Instruments. Inc.) according to the equation: *E*_RHE_ = ${{E}_{{\mathrm{Hg}}/{\mathrm{H}}{{{\mathrm{g}}}_2}{\mathrm{S}}{{{\mathrm{O}}}_4}}}\ $+ 0.64.

### *In situ* PEC Raman tests

For the *in situ* photoelectrochemical Raman (LabRAM HR Evolution, HORIBA) experiments, a 532-nm laser was used to probe and excite the surface-active species of the Ni/*n*-Si photoanode. The exposure time was set to 20 s with six-times accumulations to obtain steady-state information of surface states, and the applied potential was carried out using the CHI 1040C electrochemical workstation in a three-electrode cell with Ag/AgCl as the reference electrode. For the OER and UOR processes, the electrolyte solution was 1 M NaOH with or without 0.33 M urea.

### TPV tests

The measurements of TPV spectra were carried out using CEL-TPV2000 equipment from Beijing China Education AU-LIGHT Technology Co., Ltd. The tests were conducted in a quartz cell under an open-circuit condition with a 532-nm laser. The TPV instrument was equipped with a preamplifier, which supplied a 20-fold signal gain in our experiments. The electrolytes used were 1 M NaOH with or without 0.33 M urea, which was the same as that for the bias distribution experiments, and the reference electrode was Ag/AgCl.

## Supplementary Material

nwae053_Supplemental_File
