# Supplementary material for: Bias distribution and regulation in photoelectrochemical overall water-splitting cells
Source: Natl Sci Rev. 2024 Feb 6;11(4):nwae053. doi: 10.1093/nsr/nwae053 (PMC11044968; doi:10.1093/nsr/nwae053)
Supplement: nwae053_Supplemental_File [file nwae053_supplemental_file.docx]

**Supporting Information**

**Bias Distribution and Regulation in Photoelectrochemical Overall Water-splitting Cells**

*Kun Dang,*^1,2#^ *Siqin Liu,*^1,2#^ *Lei Wu,*^1,2^ *Daojian Tang,*^1,2^ *Jing Xue,*^1,2^ *Jiaming Wang,*^1,2^ *Hongwei Ji,*^1,2^ *Chuncheng Chen,*^1,2^ *Yuchao Zhang,*^1,2^** and Jincai Zhao*^1,2^

1 Key Laboratory of Photochemistry, CAS Research/Education Center for Excellence in Molecular Sciences, Institute of Chemistry, Chinese Academy of Sciences, Beijing 100190 (P. R. China)

2 University of Chinese Academy of Sciences, Beijing 100049 (P. R. China)

E-mail: [yczhang@iccas.ac.cn](mailto:yczhang@iccas.ac.cn)

# These authors contributed equally.

**1. Experimental section**

**1.1.** **Reagents and materials**

The *n*-Si (100) (0.05-0.20 Ω·cm, 400 μm) and *p*-Si (100) (0.001~0.005 Ω·cm, 500 μm) were purchased from the First MEMS Co., Ltd. and Hefei KeJing Materials Technology Co., Ltd., respectively. NiCl_2_·6H_2_O (99.95%) and NaClO_4_ (98%) were purchased from Alfa Aesar Chemical Co., Ltd. The H_3_BO_3_ (99.5%) and Fe(NO_3_)_3_·9H_2_O (98%) were purchased from Beijing Innochem Technology Co., Ltd., and the NaOH (99.9%) was purchased from the Shanghai Aladdin Biochemical Technology Co., Ltd. All chemicals were used as received without further purification. The anion exchange membrane (AEM) (AMI-7001) was purchased from Membranes International Inc (USA). The bipolar membrane (BPM) (Fumasep FBM-PK) was purchased from Suzhou Sinero Technology Co., Ltd. The ultrapure water with a resistivity of 18.2 MΩ·cm was obtained by purification equipment (Millipore, Milli-RO Plus).

**1.2. Materials preparation**

**Synthesis of Ni/*n*-Si photoanode.** The synthesis of Ni/*n*-Si photoanode was performed by electrodeposition according to the literature.^1^ The wafers were diced into 1 × 2 cm² rectangles and sonicated for 10 min in acetone, ethanol, and ultrapure water in sequence. Then they were sonicated for 30 min in the piranha solution (solution consisting of concentrated H_2_SO_4_ and 30% H_2_O_2_ with 1: 3 in volume), followed by washing with ultrapure water and dried by N_2_ flow. Then the ohmic contact was prepared as follows: (1) The Si surface was freshly hydrogenated by dipping it for 2 min in an HF solution (H_2_O/50% HF, V_1_/V_2_ = 5/1) and quickly dried under an N_2_ flow; (2) Scratching the top of Si surface and a droplet of In-Ga eutectic was applied on the scratched position to enhance contact, then it was covered by a layer of silver paste. After drying the silver paste (~12 h), step (1) was repeated to remove the native SiO_2_. For the electrodeposition of Ni nanoparticles, 0.1 M boric acid, and 0.1 M NiCl_2_·6H_2_O were dissolved in water in a 50 mL cell without oxygen removal, then sonicated the electrolyte for 20 min. In a three-electrode system, the cleaned Si was immersed in the electrolyte, with Pt foil (counterpart electrode) and Ag/AgCl (reference electrode). Applying −1.5 V_Ag/AgCl_ on the Si electrode for 5 s to deposit Ni nanoparticles, then the Si electrode was washed with water and dried with N_2_ flow. The capsulation of Ni/*n*-Si photoanode was carried out by epoxy glue attached to a piece of glass, and the exposed surface was 1 cm^2^.

**Synthesis of other photoanodes.** The α-Fe_2_O_3_ was grown on the fluorine-doped tin oxide substrate (FTO) through the hydrothermal and post-calcination methods, where the FeOOH was fabricated via hydrothermal process and further transformed to α-Fe_2_O_3_ by calcination in the air.^2^ The TiO_2_ (Rutile) photoanode was fabricated on the FTO substrate via a hydrothermal method with the Ti(IV) butoxide as the precursor.^3^ For the preparation of Au/TiO_2_, the as-prepared TiO_2_ photoanode was immersed into the chloroauric acid solution, and the Au/TiO_2_ was obtained by further thermal reduction.^3^ The BiVO_4_ photoanode was fabricated via the electrodeposition method in the Bi(NO_3_)_3_ solution to grow the BiOI film on the FTO substrate, then the VO(acac)_2_ solution was dropped on it following with annealing.^4^

**Characterization of Ni/*n*-Si photoanode.** The morphology of Ni/*n*-Si photoanode was characterized by the Scanning Electron Microscope (SEM, S4800, Hitachi) with 10 kV accelerating voltage. The valence states of Ni and O were obtained by the X-ray photoelectron spectroscopy (XPS, ESCALab 250Xi, Thermo Scientific) using 200 W monochromatic Al Kα radiation, where the hydrocarbon C1s line at 284.8 eV from adventitious carbon was used for calibration.

**1.3.** **Photoelectrochemical characterization**

**EIS measurements**. For EIS measurements, a sinusoidal voltage pulse of 10 mV amplitude was applied on a bias potential, with frequencies ranging from 10 kHz to 0.1 Hz, under the illumination of a white LED (100 mW cm^−2^). The raw data were fitted and simulated using Nova 2.1.4 software (Metrohm). The Mott-Schottky measurement was carried out in 1 M NaOH at 1 kHz in dark based on the Mott-Schottky equation^5^:

$\frac{1}{C^{2}} = \frac{2}{A^{2}\varepsilon_{0}\varepsilon_{r}qN_{d}} (E - E_{fb} - \frac{k_{B}T}{q})$. (1)

*C* is the differential capacitance, *A* is the surface area of the electrode, *ε_0_* is the vacuum permittivity, *ε_r_* is relative permittivity, *q* is the electronic charge, *N_d_* is the donor impurity concentration in the semiconductor, *E* is the applied potential, *𝐸_fb_* is the flat band potential, *k_B_* is the Boltzmann’s constant, and *T* is the temperature (K).

Figure S1. Three-electrode linear sweep voltammetry (LSV) curves of (a) Ni/*n*-Si, (b) α-Fe_2_O_3_, (c) BiVO_4_, and (d) TiO_2_ photoanodes. All the measurements were carried out under the illumination of AM 1.5G (100 mW cm^−2^).

Figure S2. (a) Schematic of various energy level positions in the two-electrode system consisting of the photoanode and a cathode under the short-circuit condition. (b) Variation of *φ_sc_* for Ni/*n*-Si, α-Fe_2_O_3_, BiVO_4_, and TiO_2_ under dark and illumination in two-electrode PEC cells. (c) Schematic of the band position for various semiconductors^6^. (d) Linear relation between *φ_sc_* and *V_ph_*. As a special case, the plasmonic Au/TiO_2_ was presented in Figure S4, and only semiconductors were considered here.

As shown in Figure S2a, when the photoanode and cathode are connected by a wire in ohmic contact, an equilibrium in Fermi levels of the two electrodes and the electrolyte will be established under short-circuit conditions. Such an equilibrated potential of the whole PEC cell is the physical meaning of the *φ_sc_* measured experimentally. The photogenerated electrons from the conduction bands of the photoanode travel through the external circuit to the cathode, lifting the Fermi level of the Pt cathode, as the metal Pt possesses a higher work function than these semiconductors. It was demonstrated by the control experiments as shown in Figure S2b. For the dark conditions, *φ_sc_* values of various PEC cells are almost equal to each other even with different photoanodes, which derives from the high resistances of semiconductors in the dark. In such a situation, photoanodes could be deemed as an open circuit, so the measured *φ_sc_* equals the OCP of the Pt cathode. Nevertheless, when two-electrode PEC cells were exposed to the light, all the *φ_sc_* of PEC cells shifted negatively compared with the dark conditions, indicating that the photogenerated electrons of photoanodes were transferred to the cathode.

Based on the discussion above, the position of *φ_sc_* should be related to the position of the conduction bands of photoanodes. However, no marked relationship between the two parameters was observed (Figure S2c). For example, the conduction bands minimum of BiVO_4_, Ni/*n*-Si and α-Fe_2_O_3_ possess similar positions, while their *φ_sc_* exhibits a great difference. It should be attributed to the Fermi pinning, as both Ni/*n*-Si and α-Fe_2_O_3_ have abundant surface states, which resulted in the difference in the Fermi levels of photoanodes between practical and theoretical conditions. Instead, a good linear relationship between *φ_sc_* and *V_ph_* was observed (Figure S2d), as the measured *V_ph_* was also influenced by the Fermi level pinning.

Figure S3. Investigation of ohmic potential loss (IR drop) between the Ni/*n*-Si photoanode and the Pt cathode in a single-chamber PEC OWS cell. (a) The applied *U_cell_* as function of the difference between the measured potential of the photoanode and cathode. (b) *J*–*V* curve of the PEC OWS cell.

The bias distribution of PEC OWS with various photoanodes, pH of the electrolyte and half-reactions was carried out in a single-chamber cell. To figure out the IR drop between the two electrodes in such an equipment, we compared the values of applied *U_cell_* with the difference between the measured potential of the photoanode and cathode (*φ_anode_* − *φ_cathode_*). Given that the value of IR drop is positively related to the current passing through the circuit, we carried out this experiment by using the Ni/*n*-Si photoanode due to its highest current among others. As shown in Figure S3a–b, the value of the *U_cell_* has a good linear relation with the *φ_anode_* − *φ_cathode_*, displaying a slope of 0.998, which demonstrates that the difference between them can be ignored for the condition with the current no more than 20 mA. Nevertheless, for the measurements in the H-cell, the IR drop is nonnegligible due to the membrane between the two chambers, so we separately measured the potential of the photoanode and cathode using two reference electrodes. According to the equation 1 in the manuscript, the *φ_anode_* − *φ_cathode_* rather than the applied bias (*U_cell_*) served as the denominator, which excluded the influence of the IR drop on the calculation of *PVR_anode_* for the measurements in a H-cell.

Figure S4. (a) Two-electrode bias distribution profile of the PEC OWS cell with plasmonic Au/TiO_2_ as the photoanode. (b) Three-electrode LSV curves of Au/TiO_2_. (c) *V_ph_* of Au/TiO_2_ measured by the OCP method in a three-electrode cell. The measurements were carried out under the illumination of 530 nm LED (100 mW cm^−2^). The low *V_ph_* of Au/TiO_2_ photoanode (Figure S4c) results in a relatively positive *φ_sc_* (Figure S4a), compared with PEC cells with other semiconductor photoanodes.

Figure S5. Comparison of (a) steady *J*–V curves, (b) bias distribution profiles, (c) *PVR_anode_* and (d) *PVR_cathode_* curves. The experiment was carried out in an anion exchange membrane (AEM) separated two-electrode H-cell to weaken the diffusion of constantly generated O_2_, and the electrolyte in both chambers was 1 M NaOH. For the oxygen removal experiment, the catholyte was bubbled with N_2_ flow for 25 min before the bias was applied, and it remained during the bias distribution measurements.

As a comparison, the bias distribution measurement under the oxygen removal condition was also carried out. For the cell under normal conditions (with air), a distinct current signal arose even under 0 V_cell_ as shown in Figure S5a. Similarly, the two-electrode cells with other photoanodes also presented this characteristic as shown in Figure 1b–e (manuscript) and Figure S4a. However, the non-zero currents before the OWS onset potential were not observed in the LSV curves of corresponding three-electrode cells (Figure S1), implying that it may be related to the reaction on the cathode. As shown in Figure S5a, for the oxygen removal cell with Ni/*n*-Si as the photoanode, the current was not observed until 1.1 V_cell_ (OWS), demonstrating that the current under low bias was derived from the oxygen reduction reaction (*j_ORR_*). Specifically, for Ni/*n*-Si, α-Fe_2_O_3_ and plasmonic Au/TiO_2_, the half-reaction at the photoanode involves oxidation of surface species triggered by photogenerated holes, while photogenerated electrons travel along the external circuit to proceed with ORR at the cathode.

The removal of oxygen caused a slight shift of the *φ_sc_* from 0.81 to 0.76 V_RHE_ (Figure S5b). The activation process of both photoanode and cathode were almost simultaneous, differing from the stepwise activation under the normal condition. Accordingly, a smaller value of the *PVR_anode_* was observed under low bias for the cell with oxygen removal (Figure S5c), implying that the oxygen in the catholyte shifted the onset voltage negatively (Figure S5a) via increasing the *PVR_anode_*. Although some bias was consumed by the ORR when shifting the potential of the Pt cathode negatively from *φ_sc_* to the potential of HER thermodynamics, it was negligible under high bias (> 1.0 V_cell_) as shown in Figure S5d, so more bias was indeed consumed by the HER. In addition, the influence of oxygen is inevitable in long-term electrolysis and thus the bias distribution characteristics unveiled in the cell without oxygen removal would be more general.

Figure S6. Comparison of the (a) steady *J*–V curves, (b) bias distribution profiles, (c) *PVR_anode_* curves, and (d) potential-time curves under an overall bias of 1.2 V_cell_ of TiO_2_ photoanode under conditions with air or oxygen removal. The experiment was carried out in an AEM-separated two-electrode H-cell, and the electrolyte in both chambers was 1 M NaOH. The operation for oxygen removal was the same as that in Figure S5.

As another example, the comparison with or without oxygen removal for the cell with the TiO_2_ photoanode and a Pt cathode was presented in Figure S6. Similar to the case displayed in Figure S5, the current density before the onset bias of OWS significantly decreased when the oxygen was removed in the PEC cell with TiO_2_ photoanode (Figure S6a). Actually, for photoanodes with a deep valence band (TiO_2_ and BiVO_4_, as shown in Figure S2c) in the two-electrode cell, the thermodynamics of the oxygen evolution reaction (OER) have been met under 0 V_cell_. Therefore, we attributed the non-zero current density to the coupling of OER at the photoanode and oxygen reduction reaction (ORR) at the cathode. Similar to the condition with Ni/*n*-Si photoanode, the *φ_sc_* of such a cell shifted slightly from 0.27 to 0.25 V_RHE_ (Figure S6b), which reduced the bias consumption for cathode activation. There was not much difference in the *PVR_anode_* of TiO_2_ under high bias (Figure S6c–d).

Figure S7. (a) Open-circuit potential response to the illumination of Raman laser in 1 M NaOH. (b) Photo-response to the illumination of Raman laser under different potentials in 1 M NaOH in three-electrode cell.


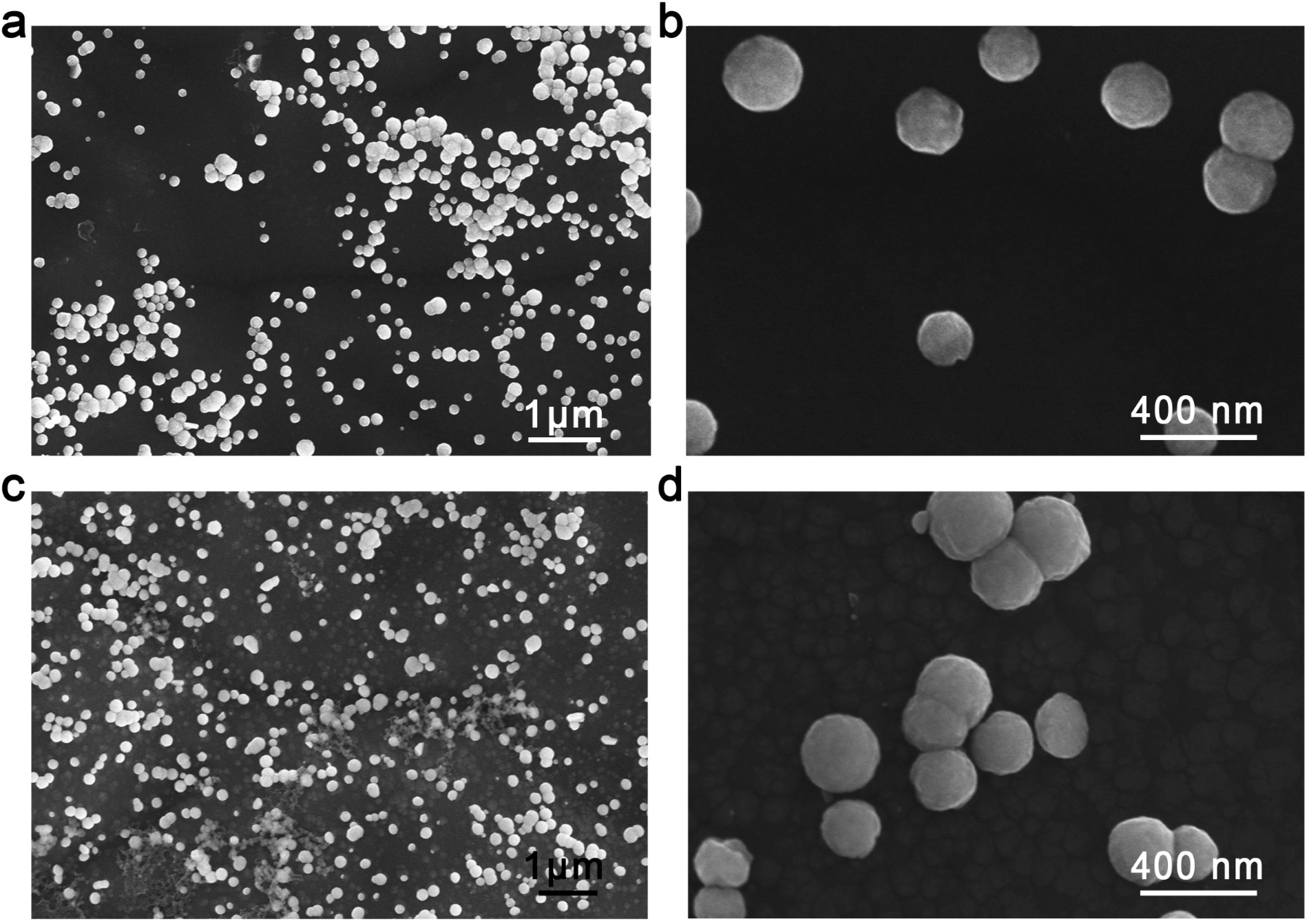


Figure S8. SEM graphics of Ni/*n*-Si photoanode (a–b) before and (c–d) after the photoelectrochemical activation via at least 30 cycles CV (0.9 ~ 2.0 V_RHE_, 0.05 V s^-1^) in the three-electrode cell. The deposited metal Ni on the surface of *n*-Si existed in the form of nanoparticles, matching well with the literature.^1^ There was no apparent change in the morphology of Ni nanoparticles after activation although the surface has transformed to Ni(OH)_2_ (Figure S9, XPS), which agreed with the previous work.^7^

Figure S9. X-ray photoelectron spectroscopy (XPS) of Ni 2p and O 1s in the Ni/*n*-Si photoanode (a and c) before and (b and d) after the PEC activation via at least 30 cycles of CV scans.

For the as-prepared Ni/*n*-Si photoanode, the peak at 851.9 and 869.3 eV were attributed to the Ni 2p_3/2_ and Ni 2p_1/2_ of metal Ni, respectively^8^. After the CV activation process, the main peaks shifted to 855.2 and 873.1 eV, corresponding to the Ni^2+^ 2p_3/2_ and Ni^2+^ 2p_1/2_,^8-9^ indicating that the metallic Ni on the surface was oxidized during the PEC process. Besides, the metal Ni^0^ signal was also detected in the Ni 2p spectra, agreeing with the core (Ni)-shell (Ni(OH)_2_) structure on the surface of the Si substrate as revealed in previous reports.^7-8^ In addition to the peak at 532.2 eV (adsorbed H_2_O) in the O 1s spectra, there were two new peaks arose at 530.6 eV and 529.0 eV for the Ni/*n*-Si photoanode after the activation, which were attributed to the metal-OH and metal-O bonds in the Ni(OH)_2_ and NiO, respectively.^10^ It demonstrated that the Ni^2+^ mainly existed as Ni(OH)_2_, matching with the results from the *in situ* Raman spectra.

Figure S10. (a) Mott-Schottky plot and (b) band structure of Ni/*n*-Si photoanode. The flat band potential of Ni/*n*-Si photoanode was estimated as 0.25 V_RHE_, which was approximate to its conduction band minimum of *n*-Si. Accordingly, the position of the valence band maximum was estimated as 1.37 V_RHE_, given the band gap of commercial *n*-Si was 1.12 eV, which agreed with the previous work^5^. Although the hole produced by the Ni/*n*-Si photoanode possesses the possibility to oxidize the H_2_O molecule due to its more positive valence band position (> 1.23 V_RHE_), it is indeed difficult as the overpotential must be considered in practice. The oxidation of OH^−^ is thermodynamically more favorable, which matched our experimental results that obvious current decay appeared in the electrolyte with lower OH^−^ concentrations (Figure 3a in the manuscript).

The pH-dependent property of photoanodes remains controversial in the PEC study. Some previous works report that the pH-dependent performance is related to quasi-Fermi-level pinning caused by surface protonation^11-12^, while it is attributed to the change in the reaction mechanism in some other works^2, 13^. For the PEC OER catalyzed by α-Fe_2_O_3_ under near-neutral conditions, the low OER activity arises from the sluggish kinetics of the proton transfer as the H_2_O is not a good proton acceptor, which can be significantly promoted by adding buffer bases^2^. For the Ni/*n*-Si photoanode, Dai’s group revealed that the OER onset potential in 1 M KOH is slightly negative compared with that in 1 M K-borate (pH = 9.5) solutions^14^, which was attributed to the higher resistance of the K-borate electrolyte. The Fermi level pinning induced by surface protonation was not deemed a crucial factor in that work.

Boettcher’s group investigated the junction behavior of Ni/*n*-Si photoanode in pH 9.8 (K-borate buffer) by using double-working electrode techniques^8^. They found that the potential shifting of *n*-Si from 0.53 to 0.98 V_RHE_ did not increase the potential of the Ni-base catalyst on the surface. Such a potential window can be deemed as the Fermi-level pinning regime. A rapid increase in the electrocatalyst potential was observed once the applied potential shifted more positively than 0.98 V_RHE_, corresponding to the transformation of Ni(OH)_2_ to NiOOH. Notably, this transformation potential of Ni species at pH 9.8 is almost identical to that at pH 13.6 in our work (Figure 2a of the manuscript), indicating that the surface protonation at pH 9.8 did not result in severe Fermi level pinning.

For the pH-dependent bias distribution measurements in a single-chamber cell in our manuscript, the electrolyte pH varied from 13.6 to 11.5. The lower limit of pH in our work is far higher than 9.8 in Boettcher’s work, so the influence of surface protonation is not a main factor for the pH-dependent performance as shown in Figure 3a–b of the manuscript. Instead, given that the *n*-Si possesses a relatively shallow valence band maximum (Figure S10a–b), the oxidation of OH^−^ rather than H_2_O is more feasible thermodynamically. Hence, the decrease of the [OH^−^] led to an obvious decrease in the OER activity. It can be further demonstrated by the reduced saturation photocurrent at relatively low pH as shown in Figure 3a of the manuscript.

Figure S11. (a) *PVR_anode_* of Ni/*n*-Si under different biases and (b) *PVR_sat_* as a function of the OH^−^ concentration in a two-electrode single cell. Comparison of (c) *J*–V curves and (d) *PVR_anode_* of Ni/*n*-Si photoanode in a two-electrode H-cell separated by an anion-exchange membrane (AEM) and bipolar membrane (BPM). For the H-cell with AEM, the electrolyte for both anode and cathode chambers were 1 M NaOH. For the H-cell with BPM, the catholyte was replaced by 0.5 M H_2_SO_4_.

To rule out the possible deviation of *φ_sc_* caused by the membrane, a control experiment was carried out where the BPM was replaced by AEM. As shown in Figure 3d in the manuscript, for the cell separated by the AEM, the *φ_sc_* was 0.81 V_RHE_, matching with the 0.89 V_RHE_ measured in a single-chamber cell with 1 M NaOH (Figure 3b). Besides, both bias-distribution curves and *PVR_anode_*–*U_cell_* curves displayed the similar shape as that measured in a single-chamber cell (Figure 3b), manifesting the reproducibility of our proposed methods for bias-distribution measurements. In contrast, for the cell with BPM, the *φ_sc_* remarkably shifted to 0.39 V_RHE_, implying that the difference in *φ_sc_* originated from the changed pH of the catholyte rather than the membrane.

Figure S12. (a) Photo-response to the illumination of Raman laser under different potentials in 1 M NaOH with 0.33 M urea, which was carried out in a three-electrode cell. (b) Comparison of photocurrents response of UOR with OER under 1.15 V_RHE_, where the photocurrent has been corrected by subtracting the dark current.

Figure S13. Potential-dependent Bode plots of Ni/*n*-Si photoanode during the (a) UOR and (b) OER process measured in the three-electrode cell.

The OER occurred at the low frequency (LF) area (Figure S13b), and a distinct peak shift was observed since 1.1 V_RHE_, which corresponded to the formation of NiOOH and the O_2_ release. As shown in Figure S13a, for the UOR process that occurred at high frequency (HF), a new peak around 10^3^ Hz arose since 0.9 V_RHE_, indicating the UOR has been triggered under such conditions. At the same time, the intensity for the peak under LF decreased, deriving from the formation and transformation of NiOOH.

Figure S14. Two configurations of the H-cell separated by the BPM under (a) forward and (b) reverse bias. The CEM refers to the cation exchange membrane, and the arrows with “*E*” refer to the electric field direction in the electrolyte.

For both configurations, the catholyte and anolyte are 0.5 M H_2_SO_4_ and 1 M NaOH, respectively. Under a forward bias (Figure S15a), the transport of ions is achieved by the migration of counter-ions of the acid (SO_4_^2−^) and alkali (Na^+^), since they can penetrate into the AEM and CEM of the BPM, respectively.^15^ Thereinto, both the HER and OER proceed in their optimal pH condition, so the theoretical bias needed to drive the OWS is reduced greatly to 0.401 V. In contrast, for the configuration under a reverse bias (Figure S15b), the transport of ions is carried out through the dissociation of the water molecules (H_2_O = H^+^ + OH^−^) under the electric field. Although the pH difference between the cathode and anode chambers remains working, i.e., the theoretical for OWS is still 0.401 V, the additional bias of 0.828 V for dissociating water must be considered, which contributes to a theoretical bias of 1.229 V for OWS.

Figure S15. (a) Bias distribution profiles of the Ni/*n*-Si photoanode and the cathode for OER||HER and OER||FRR half-reaction couplings. (b) Bias-dependent *PVR*_anode_ of Ni/*n*-Si photoanode for different half-reaction couplings. These data were obtained in a two-electrode cell.

Table S1. Electrochemical parameters fitted from the EIS data of Ni/*n*-Si during the OER in a three-electrode cell with 1 M NaOH.

| Potential  (V_RHE_) | *R_s_*  (Ω) | *R_trapping_*  (Ω) | *C_bulk_*  (μF) | *R_ct_*  (Ω) | *C_trap_*  (μF) | *χ^2^* |
| --- | --- | --- | --- | --- | --- | --- |
| 0.85 | 12.1 | 33.1 | 44.7 | 20643.0 | 17.5 | 0.0066 |
| 0.90 | 12.1 | 33.1 | 55.7 | 19372.0 | 23.6 | 0.0070 |
| 0.95 | 12.2 | 38.2 | 84.4 | 15595.0 | 35.1 | 0.0068 |
| 1.00 | 12.2 | 8.9 | 121.1 | 9822.7 | 148.9 | 0.0106 |
| 1.05 | 12.2 | 2.2 | 126.6 | 4638.0 | 315.3 | 0.0080 |
| 1.10 | 12.2 | 1.4 | 128.5 | 339.7 | 319.1 | 0.0091 |
| 1.15 | 12.3 | 0.7 | 38.9 | 40.7 | 404.6 | 0.0055 |
| 1.20 | 12.3 | 0.8 | 9.6 | 14.8 | 435.2 | 0.0007 |
| 1.25 | 12.3 | 0.4 | 13.2 | 8.0 | 455.1 | 0.0017 |
| 1.30 | 12.3 | 0.4 | 10.3 | 5.5 | 452.3 | 0.0018 |
| 1.35 | 12.3 | 0.4 | 7.8 | 4.2 | 429.4 | 0.0018 |
| 1.40 | 12.3 | 0.4 | 6.1 | 3.4 | 388.3 | 0.0018 |
| 1.45 | 12.3 | 0.5 | 4.1 | 2.8 | 335.2 | 0.0018 |
| 1.50 | 12.3 | 0.6 | 2.8 | 2.5 | 268.9 | 0.0017 |

Note: The *R_s_*, *R_trapping_*, and *R_ct_* represent the series resistance, the charge-transport resistance in the surface hole-trapping process and interfacial charge-transfer resistance, respectively. The *C_bulk_* is the space charge capacitance of the bulk, and the *C_trap_* represents the surface state capacitance.

**Reference**

(1) Loget, G.; Fabre, B.; Fryars, S.; Mériadec, C.; Ababou-Girard, S., Dispersed Ni Nanoparticles Stabilize Silicon Photoanodes for Efficient and Inexpensive Sunlight-Assisted Water Oxidation. *ACS Energy Lett.* **2017,** *2*, 569-573.

(2) Zhang, Y.; Zhang, H.; Ji, H.; Ma, W.; Chen, C.; Zhao, J., Pivotal Role and Regulation of Proton Transfer in Water Oxidation on Hematite Photoanodes. *J. Am. Chem. Soc.* **2016,** *138*, 2705−2711.

(3) Xue, J.; Wu, L.; Deng, C.; Tang, D.; Wang, S.; Ji, H.; Chen, C.; Zhang, Y.; Zhao, J., Plasmon-Mediated Electrochemical Activation of Au/TiO2 Nanostructure-Based Photoanodes for Enhancing Water Oxidation and Antibiotic Degradation. *ACS Appl. Nano Mater.* **2022,** *5*, 11342-11351.

(4) Kim, T. W.; Choi, K. S., Nanoporous BiVO_4_ Photoanodes with Dual-Layer Oxygen Evolution Catalysts for Solar Water Splitting. *Science* **2014,** *343*, 990-994.

(5) Yu, X.; Yang, P.; Chen, S.; Zhang, M.; Shi, G., NiFe Alloy Protected Silicon Photoanode for Efficient Water Splitting. *Adv. Energy Mater.* **2016,** *7*, 1601805.

(6) Mesa, C. A.; Francas, L.; Yang, K. R.; Garrido-Barros, P.; Pastor, E.; Ma, Y.; Kafizas, A.; Rosser, T. E.; Mayer, M. T.; Reisner, E.; Gratzel, M.; Batista, V. S.; Durrant, J. R., Multihole water oxidation catalysis on haematite photoanodes revealed by operando spectroelectrochemistry and DFT. *Nat. Chem.* **2020,** *12*, 82-89.

(7) Hemmerling, J. R.; Mathur, A.; Linic, S., Characterizing the Geometry and Quantifying the Impact of Nanoscopic Electrocatalyst/Semiconductor Interfaces under Solar Water Splitting Conditions. *Adv. Energy Mater.* **2022,** *12*, 2103798.

(8) Laskowski, F. A. L.; Nellist, M. R.; Venkatkarthick, R.; Boettcher, S. W., Junction behavior of n-Si photoanodes protected by thin Ni elucidated from dual working electrode photoelectrochemistry. *Energy Environ. Sci.* **2017,** *10*, 570-579.

(9) Fan, K.; Chen, H.; Ji, Y.; Huang, H.; Claesson, P. M.; Daniel, Q.; Philippe, B.; Rensmo, H.; Li, F.; Luo, Y.; Sun, L., Nickel-vanadium monolayer double hydroxide for efficient electrochemical water oxidation. *Nat. Commun.* **2016,** *7*, 11981.

(10) Chen, W.; Xu, L.; Zhu, X.; Huang, Y. C.; Zhou, W.; Wang, D.; Zhou, Y.; Du, S.; Li, Q.; Xie, C.; Tao, L.; Dong, C. L.; Liu, J.; Wang, Y.; Chen, R.; Su, H.; Chen, C.; Zou, Y.; Li, Y.; Liu, Q.; Wang, S., Unveiling the Electrooxidation of Urea: Intramolecular Coupling of the N-N Bond. *Angew. Chem., Int. Ed.* **2021,** *60*, 7297-7307.

(11) Klahr, B.; Gimenez, S.; Fabregat-Santiago, F.; Hamann, T.; Bisquert, J., Water oxidation at hematite photoelectrodes: the role of surface states. *J. Am. Chem. Soc.* **2012,** *134*, 4294-4302.

(12) Iandolo, B.; Hellman, A., The Role of Surface States in the Oxygen Evolution Reaction on Hematite. *Angew. Chem., Int. Ed.* **2014,** *53*, 13404-13408.

(13) Liu, Y.; Le Formal, F.; Boudoire, F.; Guijarro, N., Hematite Photoanodes for Solar Water Splitting: A Detailed Spectroelectrochemical Analysis on the pH-Dependent Performance. *ACS Appl. Energy Mater.* **2019,** *2*, 6825-6833.

(14) Kenney, M. J.; Gong, M.; Li, Y.; Wu, J. Z.; Feng, J.; Lanza, M.; Dai, H., High-Performance Silicon Photoanodes Passivated with Ultrathin Nickel Films for Water Oxidation. *Science* **2013,** *342*, 836-840.

(15) Ding, Y.; Cai, P.; Wen, Z., Electrochemical neutralization energy: from concept to devices. *Chem. Soc. Rev.* **2021,** *50*, 1495-1511.
